# The Prognostic Role of ST2L and sST2 in Patients Who Underwent Carotid Plaque Endarterectomy: A Five-Year Follow-Up Study

**DOI:** 10.3390/jcm11113142

**Published:** 2022-05-31

**Authors:** Pietro Scicchitano, Andrea Marzullo, Annarita Santoro, Annapaola Zito, Francesca Cortese, Cristina Galeandro, Andrea Sebastiano Ciccone, Domenico Angiletta, Fabio Manca, Raffaele Pulli, Eliano Pio Navarese, Paul A. Gurbel, Marco Matteo Ciccone

**Affiliations:** 1 Cardiology Department, Hospital “F. Perinei”, 70022 Altamura, Italy; 2 Pathology Division, Department of Emergency and Organ Transplantation, University of Bari, 70121 Bari, Italy; andrea.marzullo@uniba.it (A.M.); cardiologia1983@libero.it (A.S.C.); 3 Section of Cardiovascular Diseases, Department of Emergency and Organ Transplantation, University of Bari, 70121 Bari, Italy; annaritasantoro92@libero.it (A.S.); annapaolazito@yahoo.it (A.Z.); francescacortese@hotmail.it (F.C.); marcomatteo.ciccone@uniba.it (M.M.C.); 4 Section of Vascular Surgery, Department of Emergency and Organ Transplantation, University of Bari, 70121 Bari, Italy; cristina.galeandro@libero.it (C.G.); domenico.angiletta@uniba.it (D.A.); raffaele.pulli@uniba.it (R.P.); 5 Department of Science of Educational Psychology and Communication, University of Bari, 70121 Bari, Italy; fabio.manca@uniba.it; 6 Inova Center for Thrombosis Research and Drug Development, Inova Heart and Vascular Institute, Inova Fairfax Medical Center, Falls Church, VA 22042, USA; elianonavarese@gmail.com (E.P.N.); paul.gurbel@inova.org (P.A.G.)

**Keywords:** sST2, ST2L, carotid plaque, atherosclerosis, biomarkers, prognosis

## Abstract

Soluble suppressor of tumorigenicity (sST)-2 plasma concentration is related to atherosclerosis. The aim of this study was to assess the prognostic impact of sST2 and its membrane-associated form (ST2L) in patients with carotid atherosclerotic plaque who underwent endarterectomy (CEA). Eighty-two consecutive patients (age range: 48–86 years) who underwent CEA were enrolled. Anthropometric, clinical, instrumental, and laboratory evaluations were gathered. Thirty-seven (45%) patients were symptomatic of cerebrovascular diseases. Patients underwent a five-year follow-up. Phone calls and the analysis of national and regional databases were performed in order to evaluate the occurrence of the primary outcome (all-cause mortality). The population was divided according to survival status. Statins were administered in 81% and 87.5% of survivors and non-survivors, respectively. sST2 levels were higher in non-survivors than in survivors (117.0 ± 103.9 vs. 38.0 ± 30.0 ng/mL, *p* < 0.001) and in symptomatic individuals, compared with asymptomatic (80.3 ± 92.1 ng/mL vs. 45.4 ± 41.4 ng/mL, *p* = 0.02). ROC curve analysis identified sST2 cut-off: >98.44 ng/mL as the best predictor for mortality. At the one-year follow-up, the survival rate decreased up to 20% in patients with sST2 higher than the cut-off value. A multivariate regression analysis revealed that only sST2 (HR: 1.012, 95% CI: 1.008–1.016, *p* < 0.0001) and triglycerides plasma levels (HR: 1.008, 95% CI: 1.002–1.015, *p* = 0.0135) remained significantly associated with all-cause mortality. ST2L was not associated with all-cause mortality risk. sST2 may act as an independent prognostic determinant of all-cause mortality and symptomatic cerebrovascular diseases in patients with carotid atherosclerotic plaque who underwent CEA.

## 1. Introduction

The suppressor of tumorigenicity (ST2) protein has been described as the receptor for interleukin (IL)-33, which is a member of the IL-1 cytokine family [1]. The st2 gene is located on chromosome 2q12 and transcripts for two proteins—IL1RL1-b or ST2L, i.e., the membrane receptor member of the IL-1 receptor family, and IL1RL1-a or sST2, the truncated soluble receptor [1].

sST2 has recently been considered a reliable prognostic biomarker in patients with heart failure (HF) [2,3,4]. Specifically, it seemed to act as an independent predictor of all-cause mortality in patients with chronic HF [5,6]. Nevertheless, the role of sST2 and ST2L in atherosclerosis is less known and still controversial. Miller et al. [7] demonstrated that the IL-33–ST2L pathway might inhibit the development of atherosclerosis in apoE^−/−^ mice fed on a high-fat diet while promoting plaque development when sST2 was administered. Plasma concentrations in sST2 were related to soluble P-selectin and tissue factor (TF) expressions [8], while the concentration of ST2L in carotid plaque significantly correlated with the degree of inflammatory cells infiltration, thus promoting the vulnerability of the atherosclerotic plaques [9].

The impact of sST2 plasma concentrations and ST2L expression on the membranes of the cells of the atheroma on the overall prognosis of patients with overt atherosclerotic cardiovascular disease (ASCVD) is still a matter of debate. Willems et al. [10] observed no association between serum levels in sST2 and major adverse cardiovascular events (MACE) patients who underwent carotid endarterectomy. The KAROLA study demonstrated the association of sST2 concentration to short- and long-term all-cause mortality in patients with stable coronary heart disease [11], while a recent meta-analysis [12] reported an association between sST2 concentration and all-cause mortality in patients with the acute coronary syndrome (ACS).

The aims of this study were to (1) assess the association between sST2 serum levels concentrations and the immunohistochemical expression of ST2L in atherosclerotic plaques from internal carotid arteries of patients who underwent endarterectomy and (2) evaluate the prognostic impact of sST2 and ST2L expression on the overall survival rate of patients with ASCVD.

## 2. Materials and Methods

### 2.1. Study Population

We collected the atherosclerotic carotid plaques from 82 consecutive patients (age range: 48–86 years) who were admitted to the Vascular Surgery Section (Department of Emergency and Organ Transplantation) of the University of Bari to undergo internal carotid endarterectomy (CEA).

All patients underwent anthropometric, clinical, instrumental, and laboratory evaluations, the data of which were collected and gathered in the final dataset. Patients were enrolled between March 2016 and September 2017.

Thirty-seven (45%) patients were defined “symptomatic” in agreement with the guidelines of the European Society of Vascular Surgery (ESVS)—namely, those showing signs or symptoms of transient ischemic attack (TIA) or stroke within the last 6 months, homolateral hemispheric ischemia and/or homolateral retinal ischemia, in the absence of other embolic foci (atrial fibrillation, intracranial stenosis, etc); the presence of homolateral hemispheric lesions shown with imaging techniques (computer tomography (CT) or magnetic resonance imaging (MRI)) [13,14].

Patients who did not satisfy such characteristics were considered “asymptomatic”. Specifically, they were considered “asymptomatic” if they had no sign or symptom related to transient ischemic attack (TIA)/stroke within 6 months before study entry and showed unstable carotid plaques at imaging analysis (stenosis progression >20%, large and/or echolucent plaques, hypoechoic areas, intraplaque hemorrhage and/or necrotic core, surface irregularity, silent infarction on CT/MRI).

Cardiovascular risk factors were defined according to international guidelines [15]. Briefly, diabetes was defined as fasting plasma glucose levels of 126 mg/dL in at least two determinations, or use of antidiabetic drugs; dyslipidemia was defined as total cholesterol plasma levels >200 mg/dL, high-density lipoprotein levels <45 mg/dL, triglycerides >150 mg/dL, and/or use of lipid-lowering agents; hypertension was defined as systolic/diastolic blood pressure >140/90 mmHg [15]. Smoking habit was defined when patients assumed >10 cigarettes/die within the last 3 months. All patients underwent standard biochemical examination.

Patients underwent a five-year follow-up. Phone calls and analysis of national and regional databases were adopted in order to evaluate the occurrence of the primary outcome (all-cause mortality). Phone evaluations and rechecks of the national and regional databases were performed every six months till the end of the follow-up. All of the patients were followed up until the primary endpoint occurred.

Informed consent was obtained from each patient before enrollment. This study was performed in agreement with the ethical guidelines of the Declaration of Helsinki and approved by the Ethics Committee of the University of Bari.

### 2.2. Tissue Samples

All carotid plaques removed during CEA surgery were subjected to histological analysis. After surgical dissection, the plaques were fixed in 10% buffered neutral formalin for 24–48 h and cut into 5 mm length segments. All of them were sequentially numbered to reconstruct the entire plaque length from the proximal common carotid artery to the distal segment of the internal carotid artery and included in paraffin blocks.

Paraffin blocks were cut into 4 μm thick sections and stained with hematoxylin–eosin, Masson’s tri-chrome, and elastic fiber stains.

### 2.3. Immunohistochemistry

All of the plaque specimens were examined by immunohistochemistry for the expression of ST2L receptor in the whole section, using a rabbit polyclonal anti-ST2 antibody (IL1RL1 interleukin receptor 1, 1:500 dilution; Sigma Aldrich, St. Louis, MO, USA). The specimens were evaluated in the next days after the surgical removal. The pathologist was unaware of the clinical characteristics of the patients, and no information was according to the occurrence of the primary endpoint. All of the immunostainings were performed via an automated immunostainer (Dako Autostainer, Ft. Collins, CO, USA). Slices were pretreated with DAKO-PT-link in a pH 9 EDTA retrieval solution. Substitution of the primary antibody with PBS served as a negative control. After the preparation, the sections were simultaneously evaluated by two observers to ensure the reproducibility of the semiquantitative assessment of the plaques.

The histological examination considered both the common morphologic features of the atherosclerotic plaques—namely, fibrosis, calcification, the amplitude of the necrotic core, and the presence of complications (erosion, thrombosis, hemorrhage)—and the evaluation of the ST2L expression on the cellular component of the carotid plaques. In particular, we examined the expression of ST2L on the endothelium of the newly formed vessels. A semiquantitative method was adopted for grading the expression of ST2L on macrophages and endothelial cells. The plaques were considered negative in the absence of ST2L expression in any kind of cell (score 0–1: LOW = 0/1+). A focal positivity in cells/capillaries was graded as a moderately expressing receptor (score 2: MODERATE = 2+). A diffuse and strong expression in a large number of macrophages and capillaries was graded as high (score 3: HIGH = 3+).

### 2.4. ELISA Test

sST2 serum levels were defined using the ELISA test, and a solid-phase enzyme immunoassay was carried out to detect the presence of the ligand in a liquid sample by means of antibodies.

The ST2 ELISA kit measures soluble human ST2 protein via sandwich ELISA, with a recombinant human ST2 antibody. The assay uses two monoclonal antibodies against two different epitopes of human ST2. The ready-to-use kit is composed of the microtiter plate coated with a monoclonal antibody, peroxidase-conjugated streptavidin, and a biotinylated ST2-specific antibody. At the end of the process, the intensity of the generated color is read at 450 nm.

### 2.5. Statistics and Data Analysis

The comparisons between groups were performed with Student’s *t*-test or Mann–Whitney U test where appropriate. Receiver-operating characteristic (ROC) curve analysis was performed to calculate the area under the curve (AUC) values, and the optimal cut-off values for mortality were calculated as the point of maximum sensitivity and specificity. Survival was calculated using Kaplan–Meier analysis.

Univariate and multivariate Cox regression analyses were performed to evaluate independent predictors of mortality. *p*-values below 0.05 were defined as statistically significant. The analyses were performed using STATA software, version 12 (StataCorp, College Station, TX, USA).

## 3. Results

Table 1 gathered the main characteristics of the two groups.

All patients were on optimal medical therapy. Statins were administered in 81% and 87.5% of survivors and non-survivors, respectively, with implementation of therapies with ezetimibe in order to achieve lipid targets. Some patients (6.9% and 8.3%, respectively) were on proprotein convertase subtilisin/kexin type 9 inhibitors (PCSK9i), but no statistically significant differences were found between the two groups. Non-survivors demonstrated higher levels in leucocytes (8.4 ± 2.4 × 103/mm^3^ vs. 7.3 ± 2.0 × 103/mm^3^, *p* = 0.049), triglycerides (140.5 ± 66.4 mg/dL vs. 104.0 ± 45.6 mg/dL, *p* = 0.005) and sST2 (117.0 ± 103.9 ng/mL vs. 38.0 ± 30.0 ng/mL, *p* < 0.001) at admission.

**Figure 1:**
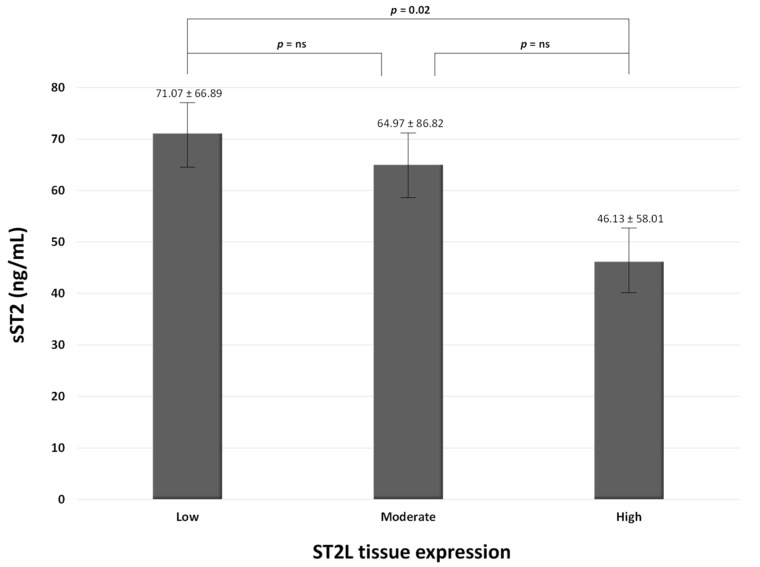
Association between the degree of ST2L expression on carotid plaques and serum levels of sST2. ns: not significant.

Figure 1 compares plasma levels in sST2 and the expression of ST2L on the surface of the cells on the carotid plaques.

In particular, the higher the expression of ST2L in carotid plaque, the lower the plasma circulating levels of sST2.

Supplementary Table S1 includes the main characteristics of asymptomatic vs. symptomatic patients. In particular, symptomatic individuals are more likely to show statistically significant increased levels in total and LDL-C, as well as in sST2 (80.3 ± 92.1 ng/mL vs. 45.4 ± 41.4 ng/mL, *p* = 0.02).

ROC curve analysis for predicting mortality identified the following cut-off: >98.44 ng/mL for sST2 and >105 mg/dL for triglycerides (Table 2).

Furthermore, ROC curve analysis for predicting symptomatic conditions revealed the following cut-offs: total cholesterol: >175.6 mg/dL, HDL-C: <55 mg/dL, LDL-C: >95 mg/dL, triglycerides: >94 mg/dL, and sT2: >95.43 ng/mL.

**Figure 2:**
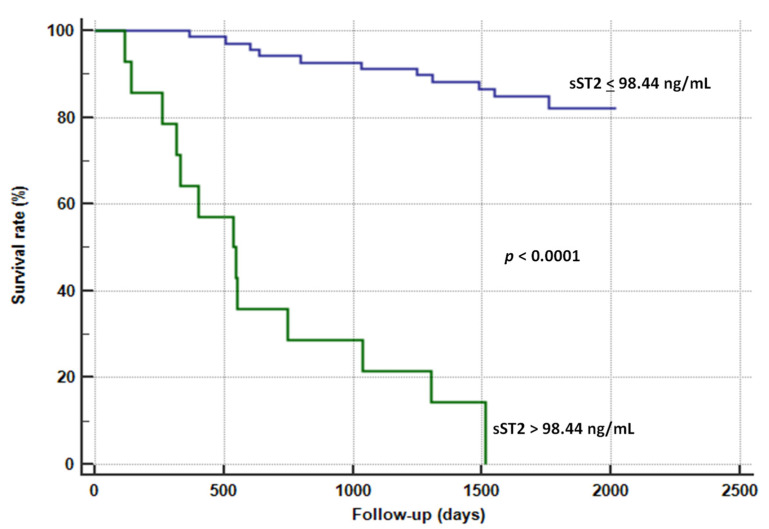
Kaplan–Meier survival curves for sST2 for the prediction of all-cause mortality.

Figure 2 outlines the Kaplan–Meier survival curves for sST2.

sST2 > 98.44 ng/mL significantly impacted survival rate: at the one-year follow-up, survival rate decreased up to 20% in patients with sST2 higher than the cut-off value.

Therefore, we evaluated the main determinants of all-cause mortality in our population in Table 3.

The multivariate regression analysis revealed that sST2 (HR: 1.012, 95% CI: 1.008–1.016, *p* < 0.0001) and triglycerides plasma levels (HR: 1.008, 95% CI: 1.002–1.015, *p* = 0.0135) significantly impacted all-cause mortality rates in our population. When splitting the population in relation to symptoms before hospital admission, we observed that sST2 plasma levels still predicted the occurrence of cerebrovascular symptoms in patients with carotid atherosclerotic plaques (HR: 1.013, 95% CI: 1.008–1.018, *p* < 0.0001) when performing multivariate regression analysis.

## 4. Discussion

The value of sST2 and its membrane-associated isoform ST2L in predicting adverse events in carotid atherosclerotic plaques is still a matter of debate. Our study demonstrated that (1) increased levels of sST2 were associated with long-term all-cause mortality and symptomatic cerebrovascular events in patients with carotid atherosclerotic plaques; (2) the presence of ST2L on the surface of atherosclerotic plaques was not able to predict adverse outcomes; (3) the higher the sST2 plasma levels, the lower the expression of ST2L on the membrane of the cells composing the carotid atherosclerotic plaques.

There is still uncertainty about the exact role of the st2 gene and its transcripts in atherosclerosis. During the last few years, researchers have focused their attention on the description of the IL-33–ST2 axis, the significance of sST2 elevation in plasma, and the role of ST2L in atherosclerotic plaques [2,3,16,17]. Nevertheless, the reciprocal correlation between these two receptors and their prognostic role in atherosclerosis and cerebrovascular diseases are still a matter of debate.

Patients at higher cardiovascular risk and with increased plasma levels in sST2 showed lower survival rates as compared with those with reduced sST2 [18,19].

Kim et al. [20] observed that patients with stable coronary artery diseases (CAD) and higher plasma levels in sST2 showed a 13.7-fold increase in major cardiovascular events (MACE)—namely, cardiac death, non-fatal myocardial infarction, coronary revascularization (90 days after ICA), and ischemic stroke. The risk for death in CAD patients with increased plasma concentration of sST2 was twofold higher as compared with those in the lowest percentiles [21,22]. Indeed, a meta-analysis from Liu et al. [12] indicated that elevated baseline sST2 levels promote a 74% increase in the long-term risk of MACE in patients with the acute coronary syndrome (ACS), thus considering a possible implication of sST2 in vulnerable plaques.

Nevertheless, the literature is scant according to the evaluation of the association among sST2/ST2L, carotid atherosclerotic plaques, and prognosis. Marzullo et al. [17] previously demonstrated that ST2L was more expressed on the membrane of macrophages and neo-vessels of carotid plaques in symptomatic patients. We observed that plaques with higher ST2L expression were related to lower plasma levels in sST2 (Figure 1). Nevertheless, ST2L expression did not provide any prognostic information in our population. We rather observed that sST2 was an independent predictor of all-cause mortality in patients with carotid plaques, as it demonstrated an AUC = 0.791, a sensibility level of 54.2%, and a specificity rate of 98.3% for a cut-off value >98.44 ng/mL (Table 2 and Figure 2). This relationship remained stable using the multivariate Cox regression analysis (Table 3). Definitely, we demonstrated that patients with higher sST2 plasma levels were more prone to die and to be symptomatic of their carotid disease. Although we observed a trend toward an inverse relationship between sST2 plasma levels and ST2L expression on the carotid atherosclerotic specimens, this correlation was lost when we performed the multivariate analysis, as well as the predictive value of the ST2L expression.

ST2L effectively failed to exert a prognostic impact in our cohort of patients, as statistical analysis did not demonstrate a predictive value for ST2L as a determinant of all-cause mortality. Similar negative results were for the discrimination between symptomatic and asymptomatic patients. We indeed believe that further analysis should be considered and performed in ad hoc clinical trials in order to better validate this relationship.

Tian et al. [23] reported an increased risk of new stroke event (ischemic or hemorrhagic) and all-cause mortality at 90-day and 1-year follow-up in patients with a transient ischemic attack (TIA)/ischemic stroke and concomitant increased plasma levels in sST2. sST2 seemed to improve the net reclassification improvement (NRI) in this specific category of patients [23]. It has been observed that increased levels in sST2 soon after stroke were related to poor outcomes (defined as modified Rankin Scale 3–6) and a 3.36-fold increase in all-cause mortality [24].

Andersson et al. [25] observed that higher sST2 plasma levels predicted subclinical brain injury, cognitive impairment, and incident stroke, thus suggesting sST2 as a potential biomarker in such a context [25,26]. Our study demonstrated that sST2 plasma level >95.43 ng/mL remained a significant predictor of symptomatic cerebrovascular diseases (Table 2).

Nevertheless, Willems et al. [10] found no correlation between sST2 plasma levels and cerebrovascular events, as well as with histopathological features of a rupture-prone carotid plaque. 

A possible explanation might be found in studies that tried to demonstrate the role of interleukin-33 (IL-33), i.e., the main ligand for ST2, on cerebrovascular diseases [27,28,29,30]. Nevertheless, further research should be performed in order to better define the relationship between the carotid plaque, ST2, and the occurrence of cerebrovascular diseases.

### Limitations

The small sample size is a limitation of this study. Nevertheless, the number of formalin-fixed, paraffin-embedded carotid plaque samples is the largest seen in the literature on this specific topic. Indeed, dedicated trials are needed in order to definitely confirm the results. Furthermore, this is a single-center study; however, a multicenter approach will more likely produce more intriguing results.

## 5. Conclusions

Plasma levels in sST2 may act as independent prognostic determinants of all-cause mortality in patients with carotid atherosclerotic plaque who underwent CEA. Increased sST2 concentrations may also discriminate symptomatic versus asymptomatic cerebrovascular diseases in patients with carotid atherosclerotic disease.

## Figures and Tables

**Table 1:** Characteristics of the study population: survivors vs. non-survivors.

Characteristics	Survivors (*n* = 58)	Non-Survivors (*n* = 24)	*p*-Values
Age (years)	71.3 ± 7.9	73.1 ± 8.6	0.36
Female (*n*/%)	38 (65.5)	20 (83.3)	0.12
Weight (kg)	78.4 ± 11.9	76.6 ± 12.9	0.55
Height (cm)	167.4 ± 7.9	166.2 ± 9.0	0.53
BMI (kg/m^2^)	28.0 ± 3.6	27.8 ± 4.4	0.84
Systolic arterial pressure (mmHg)	130.3 ± 14.4	130.6 ± 10.5	0.91
Diastolic arterial pressure (mmHg)	76.3 ± 8.0	75.8 ± 7.5	0.81
Heart rate (bpm)	63.6 ± 9.2	67.8 ± 12.6	0.09
Symptomatic (*n*/%)	23 (39.6)	14 (58.3)	0.12
Diabetes (*n*/%)	18 (31.0)	8 (33.3)	0.84
Hypertension (*n*/%)	53 (91.4)	22 (91.7)	0.97
Smokers (*n*/%)	8 (13.8)	6 (25)	0.22
Ex-smokers (*n*/%)	22 (37.9)	7 (29.2)	0.46
C-reactive protein (mg/L)	4.2 ± 3.9	5.2 ± 3.5	0.90
Leucocytes (admission) (×10^3^/mm^3^)	7.3 ± 2.0	8.4 ± 2.4	0.049
Leucocytes (peak) (×10^3^/mm^3^)	11.9 ± 4.3	11.5 ± 3.3	0.74
Total Cholesterol (mg/dL)	158.6 ± 35.3	159.4 ± 51.7	0.93
LDL-C (mg/dL)	85.9 ± 33.1	85.8 ± 42.5	0.99
HDL-C (mg/dL)	51.9 ± 19.7	45.5 ± 11.3	0.14
Triglycerides (mg/dL)	104.0 ± 45.6	140.5 ± 66.4	0.005
Troponin I (ng/mL)	0.023 ± 0.035	0.027 ± 0.028	0.61
Plaque instability features in asymptomatic pts			
- Stenosis progression > 30% (*n*/%)	9 (25.7)	3 (30)	0.79
- Large plaque (*n*/%)	10 (28.6)	6 (25)	0.07
- Necrotic core (*n*/%)	7 (20)	4 (16.7)	0.20
- Echolucent plaque (*n*/%)	7 (20)	4 (16.7)	0.20
- Hypoechoic areas (*n*/%)	6 (17.1)	4 (16.7)	0.13
- Intraplaque hemorrhages (*n*/%)	7 (20)	5 (20.8)	0.06
- Surface irregularity (*n*/%)	17 (48.6)	8 (33.3)	0.08
- Silent infarction on CT/MRI (*n*/%)	10 (28.6)	6 (25)	0.07
sST2 (ng/mL)	38.0 ± 30.0	117.0 ± 103.9	<0.001
Pharmacological treatments			
ACEi/sartans (*n*/%)	51 (87.9)	20 (83.3)	0.58
Beta-blockers (*n*/%)	42 (72.4)	17 (70.8)	0.88
Diuretics (*n*/%)	20 (34.5)	9 (37.5)	0.80
MRA (*n*/%)	11 (19.0)	5 (20.8)	0.84
Statins (*n*/%)	47 (81.0)	21 (87.5)	0.48
Ezetimibe (*n*/%)	10 (17.2)	5 (20.8)	0.71
PCSK9i (*n*/%)	4 (6.9)	2 (8.3)	0.82

Data are expressed as number ± standard deviation and/or number and percentages. Abbreviations: ACEi: angiotensin-converting enzyme inhibitors; BMI: body mass index; CT: computer tomography; HDL-C: high-density lipoprotein cholesterol; LDL-C: low-density lipoprotein cholesterol; MRA: mineralocorticoid receptor antagonist; MRI: magnetic resonance imaging; PCSK9i: proprotein convertase subtilisin/kexin type 9 inhibitors; sST2: soluble suppressor of tumorigenicity. BMI: body mass index.

**Table 2:** Performance of anthropometric, clinical, and laboratory characteristics in predicting all-cause mortality and symptomatic cerebrovascular symptoms.

**Characteristic**	**Survivors (*n* = 58)**	**Non-Survivors (*n* = 24)**	**Cut-Off**	**Sensibility**	**Specificity**	**AUC**	***p***
sST2, ng/mL	38.0 ± 30.0	117.0 ± 103.9	>98.44	54.2	98.3	0.791	<0.0001
Tryglicerides, mg/dL	104.0 ± 45.6	140.5 ± 66.4	>105	70.8	67.2	0.685	=0.0043
**Characteristic**	**Asymptomatic (*n* = 37)**	**Symptomatic (*n* = 45)**	**Cut-Off**	**Sensibility**	**Specificity**	**AUC**	***p***
Total Cholesterol, mg/dL	146.6 ± 31.6	173.7 ± 45.3	>175.6	56.8	88.9	0.722	=0.0002
HDL-C, mg/dL	54.2 ± 21.3	44.9 ± 10.5	≤55	89.2	37.8	0.628	=0.0363
LDL-C, mg/dL	70.9 ± 26.1	104.1 ± 37.9	>95	70.3	86.7	0.775	<0.0001
Tryglicerides, mg/dL	107.5 ± 54.2	123.5 ± 54.8	>94	75.7	51.1	0.623	=0.0466
sST2, ng/mL	45.4 ± 41.4	80.3 ± 92.1	>95.43	32.4	91.1	0.609	0.0855

Abbreviations: AUC: area under the curve; HDL-C: high-density lipoprotein cholesterol; LDL-C: low-density lipoprotein cholesterol; sST2: soluble suppressor of tumorigenicity.

**Table 3:** Univariate and multivariate Cox proportional hazards survival analyses and hazards for cerebrovascular symptoms related to carotid plaques.

	Univariate Cox Regression Analysis		Adjusted Cox Regression Analysis		
**Survival Analysis**					
**Parameter**	**HR (95% CI)**	***p-*** **Value**	**HR (95% CI)**	**Wald**	***p-*** **Value**
sST2, ng/mL	1.012 (1.008–1.016)	<0.0001	1.012 (1.008–1.016)	34.6856	<0.0001
Triglycerides, mg/dL	1.008 (1.002–1.015)	0.0058	1.008 (1.002–1.015)	6.0960	0.0135
**Symptomatic Cerebrovascular Diseases**					
**Parameter**	**HR (95% CI)**	***p-*** **Value**	**HR (95% CI)**	**Wald**	***p-*** **Value**
Smoking habit	2.327 (1.083–4.998)	0.0304			
Hemoglobin, g/dL	0.841 (0.716–0.988)	0.0347	0.744 (0.613–0.903)	8.9739	0.0027
HDL-C, mg/dL	0.972 (0.946–0–999)	0.0440			
LDL-C, mg/dL	1.010 (1.001–1.020)	0.0282			
Triglycerides, mg/dL	1.007 (1.001–1.013)	0.0162			
Ezetimibe	0.151 (0.021–1.104)	0.0102			
sST2, ng/mL	1.013 (1.008–1.017)	<0.0001	1.013 (1.008–1.018)	21.998	<0.0001

Abbreviations: CI: confidential index; HDL-C: high-density lipoprotein cholesterol; HR: hazard ratio; LDL-C: low-density lipoprotein cholesterol; sST2: soluble suppressor of tumorigenicity.

## Data Availability

Data are available on request by contacting the corresponding author.

## Supplementary Materials

The following supporting information can be downloaded at: https://www.mdpi.com/article/10.3390/jcm11113142/s1, Table S1: Characteristics of the study population: Symptomatic vs. asymptomatic.
