# Supplementary material for: The Prognostic Role of ST2L and sST2 in Patients Who Underwent Carotid Plaque Endarterectomy: A Five-Year Follow-Up Study
Source: J Clin Med. 2022 May 31;11(11):3142. doi: 10.3390/jcm11113142 (PMC9181783; doi:10.3390/jcm11113142)
Supplement: Supplementary file 1 [file jcm-11-03142-s001.zip › jcm-1724858-supplementary.pdf]

Supplementary Table S1. Characteristics of the study population: symptomatic vs asymptomatic.

Characteristics	Asymptomatic (n=45)	Symptomatic (n=37)	p-values
**Age (years)**	71.6 ± 8.2	72.0 ± 8.1	0.84
**Female (n/%)**	14 (31)	10 (27)	0.69
**Weight (Kg)**	77.0 ± 11.3	78.9 ± 13.2	0.47
**Height (cm)**	166.4 ± 7.8	167.9 ± 8.7	0.42
**BMI (Kg/m^2)**	27.9 ± 3.7	28.0 ± 4.0	0.88
**Systolic arterial pressure (mmHg)**	129.9 ± 14.0	130.9 ± 12.6	0.72
**Diastolic arterial pressure (mmHg)**	75.9 ± 7.6	76.5 ± 8.2	0.73
**Heart rate (bpm)**	63.6 ± 10.9	66.4 ± 9.6	0.22
**Death (n/%)**	10 (22.2)	14 (37.8)	0.12
**Diabetes (n/%)**	18 (40.0)	8 (21.6)	0.08
**Hypertension (n/%)**	44 (97.8)	31 (83.8)	**0.02**
**Smokers (n/%)**	5 (11.1)	9 (24.3)	0.12
**Ex-smokers (n/%)**	20 (44.4)	9 (24.3)	0.06
**C-reactive protein (mg/L)**	3.6 ± 1.7	5.6 ± 5.2	**0.02**
**Leucocytes (admission) (x10^3/mm^3)**	7.5 ± 1.8	7.8 ± 2.5	0.63
**Leucocytes (peak) (x10^3/mm^3)**	11.8 ± 4.5	11.8 ± 3.5	0.97
**Total Cholesterol (mg/dL)**	146.6 ± 31.6	173.7 ± 45.3	**0.002**
**LDL-C (mg/dL)**	70.9 ± 26.1	104.1 ± 37.9	**<0.001**
**HDL-C (mg/dL)**	54.2 ± 21.3	44.8 ± 10.9	**0.02**
**Triglycerides (mg/dL)**	107.5 ± 54.2	123.5 ± 54.8	0.19
**Troponin I (ng/mL)**	0.019 ± 0.019	0.031 ± 0.044	0.12
**sST2 (ng/mL)**	45.4 ± 41.4	80.3 ± 92.1	**0.02**
**Pharmacological treatments**			
**ACEi/sartans (n/%)**	42 (93.3)	29 (78.4)	**0.048**
**Beta-blockers (n/%)**	32 (71.1)	27 (73.0)	0.85
**Diuretics (n/%)**	14 (31.1)	15 (40.5)	0.38
**MRA (n/%)**	8 (17.8)	8 (21.6)	0.67
**Statins (n/%)**	40 (88.9)	28 (75.7)	0.11
**Ezetimibe (n/%)**	14 (31.1)	1 (2.7)	**<0.001**
**PCSK9i (n/%)**	5 (11.1)	1 (2.7)	0.15

Data are expressed as number ± standard deviation and/or number and percentages. Abbreviations: **ACEi:** Angiotensin-converting enzyme inhibitors; **CT:** computer tomography; **HDL-C:** high density lipoprotein cholesterol; **LDL-C:** low density lipoprotein cholesterol; **MRA:** mineralocorticoid receptor antagonist; **MRI:** magnetic resonance imaging; **PCSK9i:** Proprotein Convertase Subtilisin/Kexin type 9 inhibitors; **sST2:** soluble suppressor of tumorigenicity.
